# Identification of genes differentially expressed in association with acquired cisplatin resistance

**DOI:** 10.1054/bjoc.2000.1420

**Published:** 2000-10

**Authors:** A Johnsson, I Zeelenberg, Y Min, J Hilinski, C Berry, S B Howell, G Los

**Affiliations:** Departments of Surgery, Medicine, the Cancer Center, University of California, San Diego, La Jolla, CA, 92037-0058, USA

**Keywords:** cisplatin resistance, suppression subtractive hybridization, high throughput screening, gene expression

## Abstract

The goal of this study was to identify genes whose mRNA levels are differentially expressed in human cells with acquired cisplatin (cDDP) resistance. Using the parental UMSCC10b head and neck carcinoma cell line and the 5.9-fold cDDP-resistant subline, UMSCC10b/Pt-S15, two suppressive subtraction hybridization (SSH) cDNA libraries were prepared. One library represented mRNAs whose levels were increased in the cDDP resistant variant (the UP library), the other one represented mRNAs whose levels were decreased in the resistant cells (the DOWN library). Arrays constructed with inserts recovered from these libraries were hybridized with SSH products to identify truly differentially expressed elements. A total of 51 cDNA fragments present in the UP library and 16 in the DOWN library met the criteria established for differential expression. The sequences of 87% of these cDNA fragments were identified in Genbank. Among the mRNAs in the UP library that were frequently isolated and that showed high levels of differential expression were cytochrome oxidase I, ribosomal protein 28S, elongation factor 1α, α-enolase, stathmin, and HSP70. The approach taken in this study permitted identification of many genes never before linked to the cDDP-resistant phenotype. © 2000 Cancer Research Campaign


British Journal of Cancer (2000) 83(8), 1047–1054

© 2000 Cancer Research Campaign doi: 10.1054/ bjoc.2000.1420, available online at http://www.idealibrary.com on IDEAL®

### Identification of genes differentially expressed in association with acquired cisplatin resistance

**A Johnsson[3], I Zeelenberg[3], Y Min[1], J Hilinski[1], C Berry[3], SB Howell[2,3] and G Los[1,3]**

Departments of [1]Surgery and [2]Medicine; [3]the Cancer Center, University of California, San Diego, La Jolla CA 92037-0058, USA

**Summary** The goal of this study was to identify genes whose mRNA levels are differentially expressed in human cells with acquired cisplatin (cDDP) resistance. Using the parental UMSCC10b head and neck carcinoma cell line and the 5.9-fold cDDP-resistant subline, UMSCC10b/Pt-S15, two suppressive subtraction hybridization (SSH) cDNA libraries were prepared. One library represented mRNAs whose levels were increased in the cDDP resistant variant (the UP library), the other one represented mRNAs whose levels were decreased in the resistant cells (the DOWN library). Arrays constructed with inserts recovered from these libraries were hybridized with SSH products to identify truly differentially expressed elements. A total of 51 cDNA fragments present in the UP library and 16 in the DOWN library met the criteria established for differential expression. The sequences of 87% of these cDNA fragments were identified in Genbank. Among the mRNAs in the UP library that were frequently isolated and that showed high levels of differential expression were cytochrome oxidase I, ribosomal protein 28S, elongation factor 1α, α-enolase, stathmin, and HSP70. The approach taken in this study permitted identification of many genes never before linked to the cDDP-resistant phenotype. © 2000 Cancer Research Campaign

**Keywords**: cisplatin resistance; suppression subtractive hybridization; high throughput screening; gene expression

Cisplatin (cDDP) is one of the most widely used chemotherapeutic agents, but its effectiveness is limited by both intrinsic and acquired resistance. Acquired resistance appears to be multifactorial in that many different mechanisms participate in the defence of the cell (Los and Muggia, 1994). The mechanisms thus far identified include impaired uptake of the drug, increased efflux, intracellular detoxification by, e.g. glutathione, tolerance to the cDDP-DNA adducts and increased repair of DNA damage (Los and Muggia, 1994). The details of the biochemical steps involved have not been fully elucidated, and little information is available on how these disparate mechanisms are coordinated with each other. However, development of acquired resistance is likely to be accompanied by an altered pattern of gene expression in the cell. Changes in cDDP sensitivity have been reported in cells molecularly engineered to overexpress a variety of genes including myc, H-ras, fos, jun (Chatterjee et al, 1995), ErbB-2 (Alaoui-Jamali et al, 1997), HER-2 (Marth et al, 1997), metallothionein II (Yamada-Okabe et al, 1995), p53 (Chatterjee et al, 1995), bcl-2 (Miyake et al, 1998), and hMSH2 (Fink et al, 1996). However, whether any of these play a role in naturally-occurring acquired resistance is uncertain.

The goal of this study was to identify genes whose differential expression in cisplatin-resistant cells could be used to identify the resistant phenotype. We chose the approach of comparing a parental cell line with its isogenic subline that had been selected for acquired cDDP resistance by repeated in vitro exposure to the drug (Nakata et al, 1994). A variety of methods are now available for comparing patterns of gene expression, including differential hybridization screening (Tedder et al, 1988), subtractive library construction (Hedrick et al, 1984), representational difference analysis (RDA) (Hubank and Schatz, 1994), cDNA array hybridization (Schummer et al, 1997), serial analysis of gene expression (SAGE) (Velculescu et al, 1995) and suppression subtractive hybridization (SSH) (Diatchenko et al, 1996). The latter technique was selected for this study as it has the advantage of normalizing for mRNA abundance so that both low and high copy number mRNAs can be identified under conditions where they are differentially expressed. This approach has been recently reported to be productive in identifying differentially expressed genes in other model systems (Kuang et al, 1998; Yang et al, 1999).

In the present investigation, we used SSH to construct libraries representing mRNAs differentially expressed in the parental cDDP-sensitive human squamous cell carcinoma cell line uMSCC10b and a 5.9-fold cDDP-resistant subline. Two cDNA libraries were prepared, one containing cDNA fragments corresponding to mRNAs whose levels were increased in resistant cells (UP library), and the other containing cDNA fragments corresponding to mRNAs whose abundance was reduced in the resistant cells (DOWN library). Filter microarray hybridization was then used to document differential expression.

*Received 31 January 2000*
*Revised 19 June 2000*
*Accepted 21 June 2000*

*Correspondence to:* G Los

### MATERIAL AND METHODS

### Cells

The experiments were performed with the UMSCC10b human head and neck carcinoma cell line (Krause et al, 1981) and a variant selected in vitro with cDDP for acquired resistance (Nakata et al, 1994). This resistant variant, UMSCC10b/Pt-S15, was selected by a total of 15 repeated exposures of the parental cells to increasing concentrations of cDDP and was 5.9-fold resistant to cDDP as determined by clonogenic assay (Nakata et al, 1994). All cells were cultured in RPMI 1640 (Irvine Scientific, Santa Ana CA, USA) supplemented with 2 mM l-glutamine, 100 units $ml^{-1}$ of penicillin

G, 100 mg $ml^{-1}$ of streptomycin sulphate and 10% fetal bovine serum (Gibco BRL, Grand Island NY, USA).

### mRNA extraction

The mRNA used for library construction was isolated from 80% confluent cells by acid guanidium pheno–chloroform extraction (Chomczymski and Sacchi, 1987) followed by isolation of poly(A)+ mRNA using the Oligotex mRNA Midi Kit (Qiagen Inc, Chatsworth CA, USA). The mRNA used to make cDNA-probes directly from tumour cells was isolated by using the mRNA Direct Kit (Qiagen).

### Suppression subtractive hybridization (SSH)

SSH was performed using the ClonTech PCR-select cDNA Subtraction kit (Clontech Laboratories Inc, Palo Alto CA, USA) according to the manufacturer's instructions. Forward subtractions used cDNA fragments generated from the mRNA of the UMSCC10b/Pt-S15 subline as tester, and fragments generated from the parental UMSCC10b cells as driver. Reverse subtractions used UMSCC10b/Pt-S15 fragments as the driver. Single-stranded cDNA was made using 2 µg of mRNA from each cell line with random primers and MMLV reverse transcriptase. Double-stranded cDNA was synthesized with an enzyme-cocktail containing DNA polymerase I, RNase H, and *E. coli* DNA ligase, followed by T4 DNA polymerase. A *Rsa*I digestion was then performed to obtain shorter, blunt-ended molecules. For each subtraction, two tester populations were created by ligating two different adaptors, named 1R and 2R, onto the tester cDNA fragments. No adaptors were ligated to the driver cDNA. In a first hybridization, excess driver cDNA was mixed with tester cDNA containing adaptors 1R and 2R, respectively, in two different reactions. The tester:driver ratio was 1:100. The reactions were denatured and allowed to anneal. In a second step, these two tester-driver mixtures were hybridized together. This was followed by a primary PCR with 30 cycles and secondary PCR for 12 cycles with primers specific for the two adaptors. After the SSH procedure, theoretically only cDNA fragments that were present in greater abundance in the tester than in the driver population were equipped with both adaptors 1R and 2R. Therefore, only these fragments were exponentially amplified during the final PCR step, leading to an enrichment of the differentially expressed genes.

### TA cloning

The PCR products derived from the final SSH step were ligated into the pCR*2.1 vector by using the TA Cloning Kit (Invitrogen Co, Carlsbad CA, USA) to produce libraries of SSH-derived fragments. The ligation reaction products were then transformed into competent INVaF′ bacteria which were cultured on LB agar plates containing ampicillin and X-galactose for blue–white screening. White colonies were picked, incubated in Terrific Broth, a cocktail of bacto-tryptone (Fisher Biotech, Fair Lain NY, USA), bacto-yeast extract (Difco, Detroit MI, USA), glycerol (Fisher Biotech), KH2PO4 (Sigma, St Louis MO, USA), K2HPO4 (Sigma) and ampicillin (Fisher Biotech) and than frozen in glycerol at –80°C.

### Isolation of cDNA inserts

PCR using AmpliTaq polymerase (Perkin Elmer, Norwalk CT, USA) and nested primers directed against the inner 21 bases of adaptors 1R and 2R was performed to identify which bacterial clones contained cDNA inserts. A Perkin-Elmer Cetus DNA Thermal Cycler was programmed as follows: 94°C for 10 min to lyse the bacteria; 30 cycles of denaturation at 94°C for 30 s, annealing at 68°C for 1 min, and extension at 72°C for 1 min 30 s; final extension at 72°C for 7 min. The samples were then electrophoresed on a 1.2% agarose gel and the clones yielding a single PCR product were selected for further investigation.

### Preparation of membrane arrays

The PCR-products containing a cDNA fragment were denatured with 0.6 M NaOH and 1 µl of each fragment was dotted onto Magna Graph nylon membranes (Micron Separation Inc, Westborough MA, USA). Each membrane consisted of a maximum of 108 dots. Serial dilutions of the whole population of cDNA fragments recovered from forward or reverse SSH steps were also included in the arrays as internal controls. The membranes were neutralized with Tris-HCl and crosslinked with 120 mJ $cm^{-2}$ in a FB-UVXL-1000 UV Crosslinker (Fischer Scientific, Pittsburgh PA, USA) and stored in plastic wrap until hybridization.

### Preparation of cDNA probes

Three types of cDNA probes were used in this study. The first was PCR-amplified cDNA fragments recovered from either the forward or reverse SSH step and which putatively contained only cDNA fragment corresponding to differentially expressed mRNAs. These are referred to as forward and reversed subtracted probes. The PCR products were purified using the Advantage PCR-Pure Kit (Clontech), and the adaptors were then removed by digestion with the restriction enzymes Rsa I, Sma I and Eag I. These probes were used for the primary differential screening.

The second type of probe consisted of cDNA prepared from mRNA isolated from the two cell lines which was then PCR amplified and radiolabelled following ligation of adaptors 1R and 2R (unsubtracted PCR-amplified cDNA probes). Purification and removal of adaptors was accomplished in the same manner as for the subtracted probes. These probes were used to obtain an estimate of the degree of differential expression in cDDP-resistant vs sensitive cells.

The third type of probe consisted of cDNA from the parental UMSCC10b cell line, prepared by reverse transcription of total cellular mRNA, using the reagents of the PCR-select cDNA Subtraction Kit, that was then fragmented by digestion with Rsa I (non-amplified cDNA probes). This probe was used to study the background abundance of the gene fragments.

All probes were labelled with $^{32}P$ by utilizing the Multiprime Labeling Kit (Amersham Life Science, Arlington Heights IL, USA), with 20 ng of cDNA per probe, followed by purification with Chroma Spin-100 (Clontech) columns. The specific activity of the purified probes ranged from $5 \times 10^7$ to $8 \times 10^8$ cpm $\mu g^{-1}$ DNA.

### Array hybridization

The membrane arrays were incubated for 1 h at 68°C with 10 ml of prehybridization solution (0.2% SDS, 10 mM EDTA, 5× Denhardt's, 5× SSC, 2.5 mg salmon sperm DNA, 50 ml of blocking solution (Clonetech)) in glass hybridization tubes in a

*© 2000 Cancer Research Campaign*

Hybridization Incubator Model 400 (Robbins Scientific Co, Sunnyvale CA, USA). The radioactive probes were added and the tubes were incubated for another 16 h at 68°C. The final probe concentration in the hybridization tubes was approximately 5 ng $ml^{-1}$. The membranes were rinsed in 2× SSC, 0.2% SDS at 68°C for 4 × 20 min. Hybridizations with subtracted SSH-derived and unsubtracted PCR-amplified probes were performed in triplicate and hybridizations with non-amplified cDNA probes were performed in duplicate.

### Array imaging

Analysis of the extent of hybridization was accomplished with an imaging system from Bio-Rad Laboratories, Hercules CA, USA. Membranes were exposed to a Molecular Imaging Screen-BI in a GS-250 Sample Loading Dock for a time-period ranging from 3–12 h. The exposure time was determined empirically based on the radioactive intensity of the membranes as estimated by a Geiger–Müller counter. The exposed screens were then transferred to a Molecular Imager GS-525 and the data were analysed with the PC-based Molecular Analyst Software. A 96-circle grid with local background subtraction was applied. The three-dimensional volume analysis function was used, which gives a measure of the total signal density, including size of the dot as well as the intensity of each individual pixel. The presented values thus represent the total radioactivity per dot and are expressed as counts × $mm^2$. Due to the local background correction, some array elements yielded very low or negative values. To permit calculation of relative hybridization intensities, elements with signals of < 10 counts × $mm^2$ were assigned a value of 10 which corresponded to the visual limit of detection.

### Sequencing and identification of identified fragments

Plasmids containing cDNA fragments that were differentially expressed were sequenced using either primers homologous to the M13 reversed priming site of the plasmid, or nested primers targeted to adaptors 1R or 2R. The sequencing was performed with a 373 XL Automated DNA Sequencer (Perkin-Elmer/Applied Biosystems) at the UCSD Core Facility. The sequences were submitted for Sequence Similarity Search (BLAST search) at the GenBank of the National Center for Biotechnology Information (Internet address: http://www.ncbi.nlm.nih.gov). Fragments showing high homology ($P < 0.05$) with previously described sequences were considered to represent known genes. Fragments with high homology with more than one gene were identified on the basis of highest homology of human origin. The mRNAs for which no homology ($P > 0.05$) was found were considered unknown.

### RESULTS

### Library construction and differential screening

SSH was used to create a population of cDNA fragments corresponding to mRNAs whose levels were either increased (the UP library) or decreased (the DOWN library) in the cDDP-resistant UMSCC10b/Pt-S15 subline relative to the parental UMSCC10b cells. Figure 1 presents a flow diagram of the yield from each step of the isolation procedure. The population of subtracted cDNA fragments was ligated into a plasmid vector, and the resulting

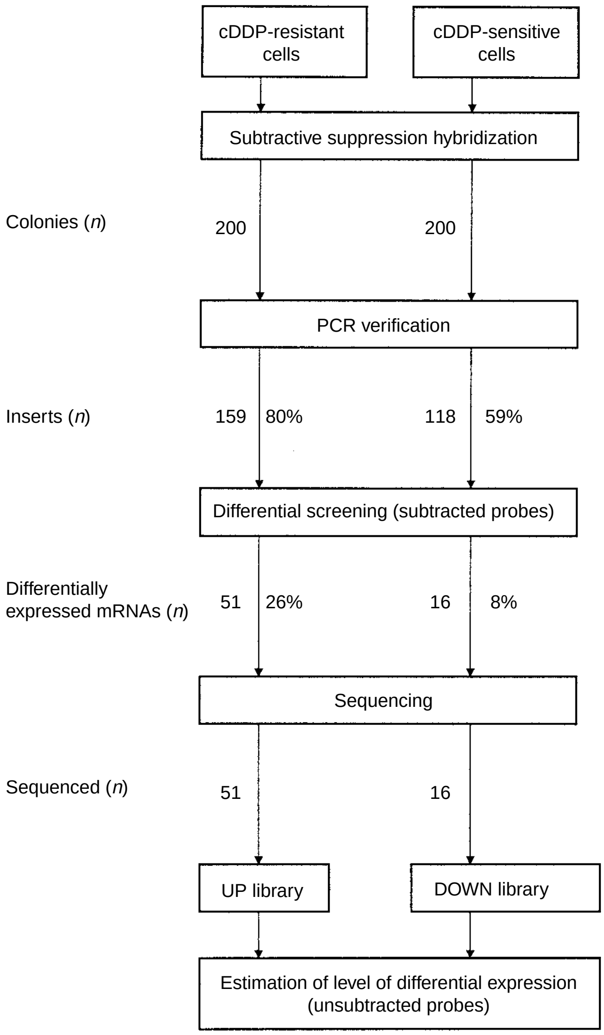

**Figure 1** Flow diagram showing the number of cDNA fragments processed for the UP and DOWN libraries libraries were transformed into bacteria. A total of 200 vector-containing bacterial colonies were picked for each library and assayed for the presence of a cDNA insert by PCR using primers specific for the adaptors ligated on either end of the insert. A single PCR product was found in 80% of the bacterial colonies from the UP library and 59% of the colonies from the DOWN library.

The PCR products generated from the inserts were arrayed on membranes, and the arrays were hybridized with forward and reversed subtracted probes, consisting of the population of cDNA fragments obtained from the SSH step from which the adaptors had been removed, to identify those elements of the array that corresponded to truly differentially expressed mRNAs. Array elements demonstrating > 5-fold differences in abundance in the UP and DOWN subtracted libraries in at least one of three repeat hybridizations were selected for further investigation. Based on this criterion, there was a clear difference in the frequency of differentially expressed cDNAs in the two libraries. Among the inserts isolated from the UP library, 47 of 159 (30%) were > 5-fold differentially represented, whereas only 16 of 118 inserts (13%) from the DOWN library met this criterion.

© 2000 Cancer Research Campaign

**Table 1** UP library: identity and differential screening, ratios of hybridization signals with forward:reversed subtracted probes, in fold-difference categories, obtained from three separate experiments

| Clone number | Fragment identity | GenBank identity | Level of differential expression | | | Function |
|---|---|---|---|---|---|---|
| | | | Exp. 1 | Exp. 2 | Exp. 3 | |
| 21 | NADH dehydrogenase | HUMMTCG | 3 | ND | 2 | Oxidative metabolism |
| 51 | Cytochrome oxidase I | HUMMTCG | 3 | 3 | 3 | |
| 50 | Cytochrome oxidase I | HUMMTCG | 3 | 3 | 3 | |
| 48 | Cytochrome oxidase I | HUMMTCG | 3 | 3 | 3 | |
| 47 | Cytochrome oxidase I | HUMMTCG | 3 | 3 | 3 | |
| 49 | Ribosomal 28S | HUMRGM | 3 | 3 | 3 | Protein synthesis |
| 42 | Ribosomal 28S | HUMRGM | 3 | ND | 3 | |
| 34 | Ribosomal 28S | HUMRGM | 3 | 3 | 3 | |
| 32 | Ribosomal 28S | HUMRGM | 3 | 3 | 3 | |
| 30 | Ribosomal 28S | HUMRGM | 3 | 1 | 2 | |
| 27 | Ribosomal 28S | HUMRGM | 2 | 3 | 2 | |
| 6 | Ribosomal 28S | HUMRGM | 2 | 3 | 1 | |
| 11 | Ribosomal S15a | HSRPS15A | 2 | 2 | 1 | |
| 28 | EF1 α | HSEF1AC | 2 | 1 | 3 | Protein synthesis, transformation, cytoskeletal organization, oncogene association |
| 25 | EF1 α | HSEF1AC | 2 | 1 | 1 | |
| 8 | EF1 α | HSEF1AC | 1 | 1 | 1 | |
| 46 | G6PDH | HSG6PDR | 2 | 2 | 3 | Metabolism, transformation resistance to radio-or chemotherapy |
| 19 | GAPDH | HUMGAPDH | 2 | 1 | 2 | Metabolism, transformation |
| 16 | GAPDH | HUMGAPDH | 2 | 2 | 1 | |
| 14 | GAPDH | HUMGAPDH | 3 | ND | 2 | |
| 43 | α-enolase | HUMENOA | 3 | 3 | 3 | Plasminogen receptor, resistance (rad or chemo) |
| 31 | α-enolase | HUMENOA | 2 | 3 | 3 | |
| 18 | α-enolase | HUMENOA | 2 | 3 | 2 | |
| 38 | Tyrosine kinase | HSTRKE | 2 | 3 | 2 | Unknown |
| 23 | PGK | HSPKG1 | 3 | 3 | 3 | Metabolism |
| 3 | Prohibitin | S85655 | 1 | 3 | 1 | Immortalization, transformation |
| 26 | Integrin α 6 | HSINTA6 | 3 | 1 | 2 | Adhesion, resistance (rad or chemo) |
| 40 | Desmoplakin | HUMDPI | 1 | 3 | 2 | Adhesion |
| 20 | Ca channel α1 | HUMCACNLS | 1 | ND | 1 | Ion transport |
| 22 | ARPF | HUMAPRF | 1 | 3 | 1 | Response to cytokines |
| 9 | Interferon γ gene | HSU10360 | 1 | 1 | 2 | Response to Interferon, protease |
| 29 | HSP70 | HSC70P | 2 | 1 | 2 | Stress response, resistance (rad or chemo) |
| 44 | Stathmin | HSRNSTATH | 1 | 2 | 3 | Oncogene association, proliferation, microtubular |
| 37 | Stathmin | HSRNSTATH | 2 | 3 | 3 | Oncogene association, proliferation, microtubular |
| 1 | GTP binding protein | HSGTPBPA | 3 | ND | 3 | Metabolism |
| 4 | GDP diss inh | HUMHRGAA | 1 | 2 | 2 | Metabolism |
| 15 | TATA-binding protein | HSU13991 | 1 | 2 | 3 | Association with oestrogen receptor |
| 17 | β-actin | HSAC07 | 1 | 2 | 1 | Cytoskeletal organization |
| 39 | p21-Arc | AF006086 | 2 | ND | 3 | |
| 35 | Keratin 6 | HUMKRT6A09 | 2 | 3 | 3 | |
| 24 | β-tubulin | HSTUB2 | 2 | ND | 2 | Microtubular function |
| 13 | β-tubulin | HSTUB3 | 1 | ND | 1 | Microtubular function |
| 41 | β-amyloid A4 | HSAPA4R | 3 | ND | 3 | Unknown |
| 45 | Unknown | | 3 | ND | 3 | |
| 36 | Unknown | | 2 | 2 | 3 | |
| 33 | Unknown | | 1 | 3 | 1 | |
| 12 | Unknown | | 1 | 2 | 2 | |
| 10 | Unknown | | 1 | 2 | 1 | |
| 7 | Unknown | | 2 | 3 | 3 | |
| 5 | Unknown | | 3 | 2 | 3 | |
| 2 | Unknown | | 1 | 2 | 1 | |

3 = > 20 fold, 2 = 5–20 fold, 1 = < 5 fold, ND = not determined; the level of differential expression refers to the ratio of hybridization signals obtained with forward and reversed subtracted cDNA probes, performed in three separate experiments; EF1α = elongation factor 1α; G6PDH = glucose-6-phosphatase dehydrogenase; GAPDH = glyceraldehyde-3-phosphatase dehydrogenase; PGK = phospho glycerate kinase; APRF = acute phase response factor; HSP70 = heat shock protein 70

In order to assess the variation between array hybridizations, the number of cDNAs meeting the 5-fold criteria was determined from each of three separate hybridizations to different copies of the same array. Tables 1 and 2 present the degree of differential expression detected by each independent hybridization. Among the 47 fragments that demonstrated a > 5-fold difference in abundance on at least one hybridization, 37 (79%) demonstrated a difference of this magnitude in at least two of the three experiments. In other words, if the 5-fold cut-off was exceeded in the first experiment for a given cDNA, there was a 79% chance that the same fragment would be scored as meeting this criterion in a least one of two additional hybridizations. Of the fragments that demonstrated a > 5-fold difference in only one of the three hybridizations, 90% still showed a difference of more than 2-fold

 *© 2000 Cancer Research Campaign*

**Table 2** DOWN library: identity and differential screening, ratios of hybridization signals with reversed: forward subtracted probes, in fold-difference categories obtained from three separate experiments

| Clone number | Fragment identity | GenBank identity | Level of differential expression | | | Function |
|---|---|---|---|---|---|---|
| | | | Exp. 1 | Exp. 2 | Exp. 3 | |
| –8 | Ribosomal L9 | HSU09953 | 2 | 1 | 1 | Protein synthesis |
| –1 | Ribosomal L10 | HUMRP10A | 2 | 2 | 1 | |
| –10 | Ribosomal L10 | HUMRP10A | 1 | 2 | 1 | |
| –13 | Ribosomal L12 | HUML12A | 1 | 3 | 3 | |
| –2 | Ribosomal L27 | HSU14968 | 3 | 3 | 3 | |
| –3 | Ribosomal L41 | AF026844 | 3 | 3 | 3 | |
| –4 | Ribosomal S3a | HUMRPSA3A | 1 | 2 | 1 | |
| –14 | Ribosomal S6 | HUMRPS6A | 2 | 2 | 1 | |
| –11 | Ribosomal S6 | HUMRPS6A | 1 | 2 | 1 | |
| –7 | Acidic ribosomal phosphoprotein | HUMPPARP0 | 1 | 1 | 2 | |
| –15 | ADP ribose polymerase | HUMPPOL | 2 | ND | 1 | Metabolism |
| –9 | Aldo-ketoreductase | HUMALRM | 2 | ND | 1 | |
| –5 | Triosephosphate isomerase | HUMTPI | 1 | 2 | 1 | |
| –6 | γ-actin | HSACTCGR | 2 | 1 | 1 | Cytoskeletal organization |
| –16 | Proliferation-associated gene | HSPAG | 2 | 2 | 1 | Proliferation |
| –12 | Unknown | | 2 | 2 | 2 | Unknown |

3 = > 20-fold; 2 = 5–20-fold; 1 = < 5-fold; ND = not determined in at least one of the two additional hybridizations. Thus, among the fragments identified as showing > 5-fold differential abundance on the first array hybridization, 46 of 47 (98%) demonstrated at least 2-fold differential expression on repeat hybridization. For these reasons, we concluded that the 5-fold cut-off applied to a single array hybridization was adequate for screening purposes.

### Identification of cDNA fragments

The cDNA fragments corresponding to the 47 mRNAs in the UP library and 16 mRNAs in the DOWN library that demonstrated > 5-fold differential expression in at least one hybridization were sequenced along with four additional fragments that also were included in the UP library, two of which had ratios of > 4.5 and two that showed ratios between 2 and 3 on all three independent hybridizations. Tables 1 and 2 show that 58 (87%) of these were identifiable as segments of cDNAs contained in GenBank, and nine (13%) were unknown. Some genes were identified more than once. mRNA encoding cytochrome oxidase I, ribosomal protein 28S, elongation factor-1α (EF-1α), glyceraldehyde-3-phosphate dehydrogenase (GAPDH), α-enolase, β-tubulin, and stathmin were identified multiple times in the UP library (Table 1), and ribosomal proteins L10 and S6 were found more than once in the DOWN library (Table 2).

### Estimation of library size

Assuming that the original cDNA library represented all mRNAs expressed in the cell, since the SSH technique normalizes the abundance of the mRNAs, an estimate of the number of mRNAs that are differentially expressed between the parental UMSCC10b and UMSCC10b/Pt-S15 cells can be made from the number of duplicates recovered. Of the 51 fragments in the UP library, the gene to which these corresponded could be identified in 43. Of these 43, 19 were recovered once, two were recovered twice, three three times, one four times, and one seven times. The method of Chao (1987) yielded an estimate of 116 cDNAs whose cognate gene could be identified in GenBank in the UP library (95% CI 47–409). To accommodate the fact that the cognate gene could not be identified for some of the differentially represented cDNAs, this estimate should be increased by a factor of 51/43, resulting in an adjusted estimate of 138 genes. Of the 16 fragments in the DOWN library, 15 were identified as belonging to sequences in Genbank. Eleven were recovered only once and two were recovered twice. This yields an estimated library size of 43 (95% CI 19–158). The estimate adjusted for cDNAs whose cognate gene could not be identified in GenBank is 46. These calculations suggest that approximately 25% of the mRNAs that are actually differentially expressed in the UMSCC10b and UMSCC10/Pt-S15 cells are represented in the subtracted libraries. However, the estimates of the library size are based on relatively small numbers of cDNAs isolated more than once and values as low as 10% or as high as 50% are consistent with these data.

### Magnitude of differential expression determined by reverse Northern blot analysis

The magnitude of the difference in abundance between the cDDP-sensitive and -resistant cells in the 51 cDNAs included in the UP and the 16 cDNAs of the DOWN libraries was examined further by reverse Northern blotting. cDNA was prepared from the mRNA of the sensitive or resistant cells, adaptors were ligated, and the cDNA population PCR amplified, radiolabelled and used to probe the filter arrays. These hybridizations were repeated three times and the mean level of differential expression for each element in the array was calculated as the ratio between resistant and sensitive cells (Figure 2). Among the 51 cDNAs included in the UP library, 40 (78%) corresponded to mRNAs that demonstrated at least a 1.7-fold difference in level, 18 (35%) at least a 5-fold difference, 12 (20%) a > 10-fold difference, and eight (16%) a > 20-fold difference. Particularly high ratios were observed for mRNAs encoding cytochrome oxidase I, ribosomal protein 28S, glucose-6-phosphate dehydrogenase G6PD), stathmin and unknown clone #45. Among the 16 cDNAs in the DOWN library, seven (44%) demonstrated at least a 1.7-fold difference in expression and three (19%)

© 2000 Cancer Research Campaign

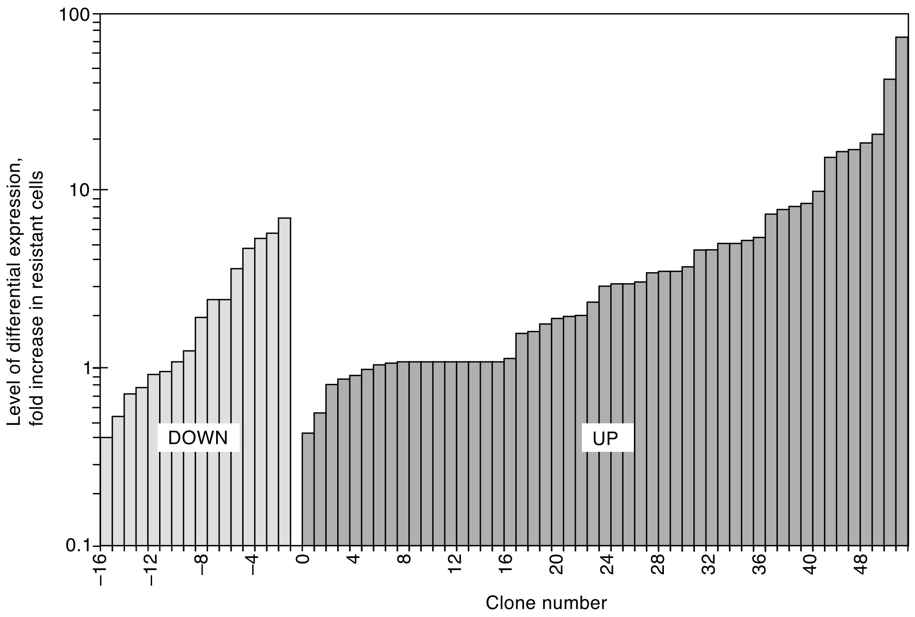

**Figure 2** Distribution of the ratios of mRNA level in the cDDP-resistant UMSCC10b/15S to that in the -sensitive UMSCC10b cells for each cDNA clone in the UP and DOWN library, respectively. Hybridizations were performed with unsubtracted PCR-amplified probes. Each bar represents the mean value of three experiments. The identity of each clone is given in Tables 1 and 2 along with the individual clone number at least a 5-fold difference. None had a > 10-fold difference. Six (38%) of the fragments had a ratio of < 1.0, suggestive of a down-regulation in the resistant cell line.

### Abundance of differentially expressed mRNAs

The distribution of the absolute abundance in the parental UMSCC10b cells of each differentially expressed mRNA provides a test of the ability of the SSH technique to recover low vs high abundance transcripts. The absolute abundance of the mRNA corresponding to each cDNA fragment meeting the criteria for differential expression was estimated from analysis of the hybridization signal obtained by probing the arrays with non-amplified cDNA prepared by reverse transcription from total mRNA harvested from the UMSCC10b cells. The mRNAs were arbitrarily categorized as being of low (< 10 counts $\times$ mm$^2$, i.e. below the visual detection limit), medium (10–100 counts $\times$ mm$^2$), and high (> 100 counts $\times$ mm$^2$) abundance. Results from array elements corresponding to mRNAs of the same identity were averaged together. In the UP library, 66% of the mRNAs were of low abundance, 25% of medium and 9% of high abundance. In the DOWN library 7%, 64%, and 29% of the fragments were in the low, medium, and high abundance categories, respectively. Thus, most of the mRNAs whose level was increased in the resistant cells were of low abundance, whereas the majority of the mRNAs whose level was decreased were of medium or high abundance.

### DISCUSSION

In the present study we combined a PCR-based subtraction strategy with cDNA array hybridization to identify mRNAs differentially expressed in a single isogenic pair of cDDP-sensitive and -resistant cells. In this pair, whose resistant phenotype has been stable over many generations, the resistant phenotype was found to be accompanied by changes in the level of numerous mRNAs, most of which have never been linked to cDDP resistance before. Studies of differential gene expression have often been performed with techniques such as RT-PCR and Northern blotting, which both have the disadvantage of permitting simultaneous analysis of only a very limited number of genes. The present study demonstrated that the approach of enriching for differentially expressed mRNAs using the SSH technique followed by analysis of the recovered fragments on cDNA arrays was reasonably efficient in identifying differentially expressed genes. Twenty-six percent of the fragments from the UP library and 8% from the DOWN library corresponded to mRNAs that differed in abundance by > 5-fold on at least one of three repeat array hybridizations. Although modest, these percentages are of the same order of magnitude as a recent study by Yang et al (1999) who found that 23% of the 332 clones were differentially expressed in oestrogen receptor-positive compared to -negative cells.

The reproducibility of the membrane arrays was reasonably good. There was a substantial numerical variation in the hybridization signals between the arrays, but among the fragments demonstrating a > 5-fold increase in one of the hybridizations, 90% showed a difference of at least 2-fold in at least one of the two additional hybridizations. This reproducibility was considered good enough for screening purposes.

The SSH technique includes a step directed at normalizing the abundance of different cDNAs to facilitate the identification of mRNAs that are differentially expressed but whose absolute levels are too low to be detected by Northern blot analysis. The results of the present study demonstrate that the SSH technique was efficient in recovering such mRNAs. Half (50%) of the differentially expressed transcripts were below the limit of detection when

 *© 2000 Cancer Research Campaign*

hybridized with cDNA produced from the mRNA of the parental UMSCC10b and radiolabelled without any PCR amplification. The issue of whether microarrays are better or worse than Northern blot analyses for quantification of mRNA level remains unresolved, but is in any case moot for many of the mRNAs identified in this study because of their low abundance.

The difference in level of expression was modest for most of the identified mRNAs. However, for 20% of mRNAs in the UP library the difference was > 10-fold. The finding that the majority of mRNAs show little change, and a progressively smaller fraction shows incrementally larger changes, is consistent with results obtained in other systems where isogenic cells growing under different conditions have been compared (Zhang et al, 1997; Zhou et al, 1998).

Based on the number of duplicates recovered in the UP and DOWN libraries, it was estimated that 138 mRNAs were up-regulated in the cDDP-resistant cells (95% CI 50–410) and 46 were down-regulated (95% CI 20–160). These estimates are of the same order of magnitude as those made for the number of differentially expressed mRNAs in other isogenic comparisons (Zhang et al, 1997; Zhou et al, 1998). When the SAGE technique was used to examine the levels of 45 000 mRNAs in colon cancer cells vs normal colon epithelium, 289 transcripts were found to be differentially expressed, 181 down- and 108 up-regulated (Zhang et al, 1997). A comparison of normal and malignant pancreatic cells using the same technique identified 183 transcripts whose expression were significantly elevated in the cancer cells (Zhou et al, 1998). The estimated fraction of transcripts exhibiting significant differences in expression in the cDDP-sensitive vs-resistant cells was between 0.25 and 1%, and this is close to the estimate of 1.5% made for normal vs malignant colon and pancreatic epithelial cells (Zhang et al, 1997; Zhou et al, 1998). Thus, it appears that acquired cDDP-resistance was accompanied by changes in only a small fraction of all transcripts expressed in the parental cells.

The goal of this study was to identify mRNAs that might be useful in diagnosing the cDDP-resistant phenotype rather than documenting that any of them were in fact causative of cDDP resistance. It is unlikely that the mRNAs changes identified in this study were simply due to clonal variation, since we compared entire cDDP-sensitive and -resistant populations rather than individual clones. Many of the changes observed may be secondary effects of the primary causative genetic changes that produce the resistant phenotype. Nevertheless, such changes can be useful markers of the cDDP-resistant phenotype, and among the changes identified, several stand out as particularly interesting candidates for investigation using additional pairs of cDDP-sensitive and -resistant cell lines. Cytochrome oxidase I is a good example due to its very high level of upregulation in resistant cells, and to the fact that it was isolated four times in the UP library. Increased cytochrome oxidase I activity has been demonstrated in cDDP-resistant variants of the MCF-7, 2008 and SCC-25 cell lines (Ara et al, 1994). Mitochondria play a central role in apoptosis (Green and Reed, 1998), and other studies have shown changes in mitochondrial membrane potential when tumour cells become resistant to cDDP (Andrews and Albright, 1992).

### ACKNOWLEDGEMENTS

This study was supported in part by grants RO1 CA77618 from the National Cancer Institute and RPG-99-159-01 from the American Cancer Society and was conducted in part by the Clayton Foundation for Research – California Division. Drs Howell and Los are Clayton Foundation Researchers.

### REFERENCES

Alaoui-Jamali MA, Paterson J, Moustafa AE and Yen L (1997) The role of ErbB-2 tyrosine kinase receptor in cellular intrinsic chemoresistance: mechanisms and implications. *Biochem Cell Biol* **75**: 315–325

Andrews PA and Albright KD (1992) Mitochondrial defects in cis-diamminedichloroplatinum(II) resistance in human ovarian carcinoma cells. *Cancer Res* **52**: 1895–1901

Ara G, Kusomota T, Korbut TT, Cullere-Luengo F and Teicher BA (1994) Cis-diamminedichloroplatinum(II) resistant human tumor cell lines are collaterally sensitive to $PtCl_4(Rh\text{-}123)_2$: evidence for mitochondrial involvement. *Cancer Res* **54**: 1497–1502

Chao A (1987) Estimating the population size for capture-recapture data with unequal catchability. *Biometrics* **43**: 783–791

Chatterjee D, Liu CJ, Northey D and Teicher BA (1995) Molecular characterization of the in vivo alkylating agent resistant murine EMT-6 mammary carcinoma tumors. *Cancer Chemother Pharmacol* **35**: 423–431

Chomczymski P and Sacchi N (1987) Single step method of RNA isolation by acid guanidium phenol-chloroform extraction. *Anal Biochem* **162**: 156–159

Diatchenko L, Lau YFC, Campbell AP, Chenchik A, Moqadam F, Huang B, Lukyanov S, Lukyanov K, Gurskaya N, Sverdlov ED and Siebert PD (1996) Suppression subtractive hybridization: a method for generating differentially regulated tissue-specific cDNA probes and libraries. *Proc Natl Acad Sci USA* **93**: 6025–6030

Fink D, Nebel S, Aebi S, Zheng H, Cenni B, Nehme A, Christen RD and Howell SB (1996) The role of DNA mismatch repair in drug resistance. *Cancer Res* **56**: 4881–4886

Green DR and Reed JC (1998) Mitochondria and apoptosis. *Science* **281**: 1309–1321

Hedrick SM, Cohen DI, Nielsen EA and Davis MM (1984) Isolation of cDNA clones encoding T cell-specific membrane-associated proteins. *Nature* **308**: 149–153

Hubank M and Schatz DG (1994) Identifying differences in mRNA expression by representational difference analysis of cDNA. *Nucleic Acids Res* **22**: 5640–5648

Krause CJ, Carey TE, Ott RW, Hurbis C, McClatchey KD and Regezi JA (1981) Human squamous cell carcinoma: establishment and characterization of new permanent cell lines. *Arch Otolaryngol* **107**: 703–710

Kuang WW, Thompson DA, Hoch RV and Weigel RJ (1998) Differential screening and suppression subtractive hybridization identified genes differentially expressed in an estrogen receptor positive breast carcinoma cell line. *Nucleic Acids Res* **26**: 1116–1123

Los G and Muggia FM (1994) Platinum resistance; experimental and clinical status. *Hematol Oncol Clin North America* **8**: 411–429

Marth C, Widschwendter M, Kaern J, Jürgensen NP, Windbichler G, Zeimet AG, Trope C and Daxenbichler G (1997) Cisplatin resistance is associated with reduced interferon-gamma-sensitivity and increased HER-2 expression in cultured ovarian carcinoma cells. *Br J Cancer* **76**: 1328–1332

Miyake H, Hanada N, Nakamura H, Kagawa S, Fujiwara T, Hara I, Eto H, Gohji K, Arakawa S, Kamidono S and Saya H (1998) Overexpression of Bcl-s in bladder cancer cells inhibit apoptosis induced by cisplatin and adenviral-mediated p53 gene transfer. *Oncogene* **16**: 933–943

Nakata B, Barton RM, Robbins KT, Howell SB and Los G (1994) Association between hsp60 mRNA levels and cisplatin resistance in human head and neck cancer cell lines. *Int J Oncology* **5**: 1425–1432

Schummer M, Ng W, Nelson P, Bumgarner R and Hood L (1997) Inexpensive handheld device for the construction of high-density nucleic acid arrays. *Biotechniques* **23**: 1087–1092

Tedder TF, Streuli M, Schlossman SF and Saito H (1988) Isolation and structure of a cDNA encoding the B1 (CD20) cell-surface antigen of human B lymphocytes. *Proc Nat Acad Sci USA* **85**: 208–212

Velculescu VE, Zhang L, Vogelstein B and Kinzler KW (1995) Serial analysis of gene expression. *Science* **270**: 484–487

Yamada-Okabe T, Yamada-Okabe H, Kashima Y and Doi R (1995) Effects of oncogenes on the resistance to cis-diamminedichloroplatinum II and metallothionein gene expression. *Toxicol Appl Pharmacol* **133**: 233–238

© 2000 Cancer Research Campaign 

Yang GP, Ross DT, Kuang WW, Brown PO and Weigel RJ (1999) Combining SSH and cDNA microarrays for rapid identification of differentially expresses genes. *Nucleic Acids Res* **27**: 1517–1523

Zhang L, Zhou W, Velculescu VE, Kern SE, Hruban RH, Hamilton SR, Vogelstein B and Kinzler KW (1997) Gene expression profiles in normal and cancer cells. *Science* **276**: 1268–1272

Zhou W, Sokoll L, Bruzek DJ, Zhang L, Velculescu VE, Goldin SB, Hruban RH, Kern SE, Hamilton SR, Chan DW, Vogelstein B and Kinzler KW (1998) Identifying markers for pancreatic cancer by gene expression analysis. *Cancer Epidemiol Biomarkers Prev* **7**: 109–112

 *© 2000 Cancer Research Campaign*
